# Recent advances in the first-line treatment of follicular non-Hodgkin lymphoma

**DOI:** 10.12688/f1000research.16686.1

**Published:** 2019-03-15

**Authors:** Pierre Feugier, Lauriane Filliatre-Clement

**Affiliations:** 1 Department of Hematology, CHRU Nancy Brabois, Vandoeuvre les Nancy, France; 2 Unité INSERM 1256, Faculté de Médecine, Université de Lorraine, Lorraine, France

**Keywords:** follicular lymphoma, treatment, first line

## Abstract

Follicular lymphoma (FL) is the most common type of indolent B-cell lymphoma. Twenty years ago, FL was considered an indolent lymphoma with a long survival time but without a high rate of complete remission with chemotherapies. The use of rituximab has improved the response and survival of patients with this lymphoma. More recently, development of biological knowledge and use of targeted drugs have offered new perspectives, including improvement of response rates and survival with chemo-free treatment strategies. In 2019, patients have a 10-year overall survival probability of over 70%. Histological transformation to more aggressive lymphoma and treatment relapses remain a medical challenge, especially for patients relapsing within two years. This article will review the recent advances in the treatment of FL. As the use of new drugs is directly related to the development of biological aspects, we will first summarize recent advances in biological aspects of FL.

## Biology

Follicular lymphoma (FL) typically causes clonal proliferation of neoplastic lymphoid cells with molecular genetic traits, morphology, and immunophenotype similar to those of germinal center B cells ^1^. According to the World Health Organization, FL ought to be categorized into histological grades (1 to 3) according to the quantity of centroblasts per high-power field and whether centrocytes are present (3A or 3B). Grade 3B FLs are usually excluded from FL clinical trials and are considered a more aggressive lymphoma. FL is characterized by the reciprocal translocation t(14;18)(q32;q21), which is present in 85 to 90% of cases. It is the first step of lymphomagenesis, occurring within the bone marrow during B-cell lymphopoiesis. The t(14;18) translocation means that the B-cell lymphoma 2 ( *BCL2*) gene is influenced by transcriptional enhancers associated with immunoglobulin heavy locus (IGH); it results in overexpression of anti-apoptotic BCL2 and brings about improved cell survival and uncontrolled cell proliferation in germinal centers via overexpression of the anti-apoptotic BCL2 protein. This rearrangement is probably insufficient for malignant transformation since it is observed at low frequency in the peripheral blood of more than 50% of healthy individuals ^2^; secondary genetic alterations are required for cellular transformation to FL. *BCL2* is also frequently mutated in FL, and *BCL2* mutations were recently shown to be associated with an increased risk of transformation and risk of death due to lymphoma ^3^. FL pathobiology is complicated by sweeping somatic changes occurring in both the genome and the epigenome, as indicated by frequent mutations in chromatin-modifying genes, such as KMT2D and CREBBP. A number of cellular pathways, including BCL6, mTOR, TNFRSF14, and JAK-STAT, are also altered. Gene expression profiling studies in FL demonstrate that the tumor microenvironment is an important determinant of outcome. Genes expressed by non-tumoral cells, especially T cells and macrophages, appear to be important predictors of outcome. Generally, an increased T-cell number is correlated with a positive prognosis whereas an increased number of macrophages is associated with progression and an unfavorable prognosis in patients with FL ^4^. However, this poor prognosis can be circumvented by the use of rituximab ^5^. The discovery of the role of the microenvironment in FL led to the use of new drugs targeting the immune system, including immunomodulatory drugs (that is, lenalidomide) and immune checkpoint inhibitors (that is, ipilimumab and pidilizumab).

## Prognosis

The Follicular Lymphoma International Prognosis Index (FLIPI) classified patients with FL into three groups according to overall survival (OS). Five adverse prognostic factors were selected—age, Ann Arbor stage, hemoglobin level, number of nodal areas, and serum lactate dehydrogenase level—leading to the definition of three risk groups related to OS ^6^. Given that a long period of time is needed for OS to be assessed, the FLIPI-2 index was developed with progression-free survival (PFS) as the primary end point and was based on a series of patients who received anti-CD20 monoclonal antibody ^7^. This index relies on five different prognostic parameters: longest diameter of the largest tumor mass greater than 6 versus less than 6 cm, serum beta-2 microglobulin level (higher versus lower limit of normal), bone marrow involved or not, hemoglobin level greater than 120 versus less than 120 g/L, and age greater than 60 versus less than 60 years. Despite the utility of FLIPI for prognosis, treatment initiation in patients with FL is decided by assessment of staging and tumor burden with the Groupe d’Etude des Lymphomes Folliculaires (GELF) ^8^ criteria while taking into account the presence of B symptoms, cytopenias, or size of the tumor. Finally, Bachy *et al*. recently reported that bone marrow involvement and beta-2 microglobulin could predict PFS and also isolated patients progressing in the first two years post-immunochemotherapy (PRIMA-PI) ^9^.

FLIPI-1 and -2 indexes are used to predict survival but are not accurate enough to identify a group of patients with a bad prognosis. New biological models predicting poor outcomes prior to treatment failure have recently been reported. Huet *et al*. ^10^ developed a score based on biological characteristics looking at 23 genes reflecting both B-cell biology and tumor microenvironment, and also predicted PFS. In multivariate analysis, the score predicted PFS independently of anti-CD20 maintenance and of the FLIPI score, which could help in providing personalized therapies tailored to at-risk patients. However, it could not be used routinely in all labs now but is available from a formalin-fixed biopsy ^10^.

Finally, the M7-FLIPI combines the mutation status of several genes with FLIPI score to improve the identification of FL patients at high risk of progression in a new clinical and genetic model. It combines FLIPI score and ECOG (Eastern Cooperative Oncology Group) performance status with the mutation status of seven genes ( *EZH2, ARID1A, MEF2B, EP300, FOXO1, CREBBP, and CARD11*). It defines a high-risk group as having a 5-year failure-free treatment of 25% and a low-risk group as having a 5-year failure-free treatment of 68% ^11^.

Other prognostic scores have been developed based on the fact that patients who have a progression of disease within 24 months (POD24) have a worse prognosis. Indeed, a study by Casulo *et al*. ^12^ of 558 patients found a lower 5-year OS of 50% for patients with POD24 who received an R-CHOP (rituximab plus cyclophosphamide, doxorubicin, vincristine, and prednisone) regimen versus 90% for patients without POD24. PRIMA-PI and M7-FLIPI have also been reported to be predictive of POD24.

## Assessment of positron emission tomography scan

Histological transformation occurs in 5 to 10% of patients and carries a poor prognosis. Positron emission tomography (PET) scans guide diagnostic biopsy in the more metabolic site. PET scans could also be used for prognostication at baseline and evaluation at the end of treatment of the FL. At the end of first-line therapy, PET scans could predict risk of relapse. In a multi-center retrospective review, the hazard ratio (HR) for PFS for patients with a positive PET scan versus those with a negative PET scan was 3.9 (95% confidence interval [CI] 2.5–5.9; *P* <0.0001) and the HR for OS was 6.7 (95% CI 2.4–18.5; *P* = 0.0002) ^13^. Recently, a model incorporating these two factors was built. Their combination stratified the population into three risk groups with 5-year PFS rates of 67%, 33%, and only 23%, respectively ^14^. More recently, the GALLIUM trial, which showed that patients with FL had a longer PFS after first-line immunochemotherapy with obinutuzumab than with rituximab, investigated the role of PET scans at the end of treatment ^15^. According to Lugano 2014 criteria, 2.5-year PFS rates were 87.4% (95% CI 83.7–90.2%) in complete metabolic responders and 54.9% (40.5–67.3%; HR 0.2, 95% CI 0.1–0.3, *P* <0.0001) in non-complete metabolic responders ^16^.

## Treatment of low-burden follicular lymphoma

With localized and low-burden FL, clinical studies are old and rare, include heterogeneous populations (staging, FLIPI, and type of treatment), and have contradictory results. Moreover, the recent use of PET scans could improve the quality and precision of staging, limiting the number of real localized low-burden FL. If radiotherapy is an option, then watching and waiting (WW) is the rule.

But the majority of low-burden FLs are not localized. Before the rituximab era, observation was the gold standard. Evidence from different groups showed that early chemotherapy held no benefit for patients. The British lymphoma pathology group compared oral chlorambucil versus observation in patients with low-burden FL, and there was no difference in terms of OS with a median follow-up of 16 months. The proportion of patients not needing chemotherapy at 10 years was 19% ^17^. The same group evaluated the role of rituximab in delaying the need of treatment (chemotherapy or radiotherapy) and its role in quality of life (QoL); in a total of 379 patients, low–tumor burden FL was treated by WW, rituximab 375 mg/m² every week for 4 weeks (rituximab induction), or rituximab induction with a subsequent maintenance schedule (12 infusions given every 2 months over 2 years) in a phase 3 trial ^18^. QoL at month 7 and time to start of new treatment were the primary end points. In addition to PFS, a notable difference in the time to start of new treatment was found: at the 3-year point, 46% of patients in the WW group did not need treatment in comparison with 88% of those in the maintenance group. Between the maintenance and induction groups, there was no reported difference. Finally, the RESORT (Rituximab Extended Schedule or Re-treatment) study evaluated the role of rituximab maintenance (MR) (that is, rituximab every 3 months) in a re-treatment rituximab (RR) strategy. RR patients were re-treated at each progression until treatment failure. With a median follow-up of 4.5 years, the estimated median time to treatment failure was not significantly different between the RR group (3.9 years) and MR patients (4.3 years) ( *P* = 0.54) ^19^.

To conclude, in low-burden FL treatment, the WW strategy continues to be the gold standard but four rituximab infusions could be used as an alternative; MR was not an effective strategy in this situation. In regard to the RR strategy, RR was shown to be possible at each disease progression until failure of the treatment.

## Advanced-stage symptomatic disease is treated with rituximab and chemotherapy followed by rituximab maintenance

Moderate improvements in FL OS had been shown before rituximab was introduced and these depended on a number of factors, including the use of interferon, autologous stem cell transplantation, and supportive care ^20, 21^. The efficacy, in terms of improvements in PFS and OS, of rituximab in combination with a different chemotherapy has been shown in four randomized trials since 2005. Marcus *et al*. investigated the use of rituximab plus cyclophosphamide, vincristine, and prednisone (R-CVP) compared with CVP in 321 patients and showed a significantly improved 4-year OS (83% versus 77%, *P* = 0.029) ^22^. Hiddemann *et al*. found similar results by comparing R-CHOP and CHOP in a total of 428 patients ^23^. Herold *et al*. randomly assigned patients to a regimen of mitoxantrone, chlorambucil, and prednisolone (MCP) chemotherapy plus rituximab or MCP alone; an improvement in median PFS and OS was observed (PFS, not reached versus 28.8 months, respectively; *P* = 0.0001, 4-year OS rate, 87% versus 74%, respectively; *P* = 0.0096) ^24^. Finally, the French GELA (Groupe d’Etude des Lymphomes de l’Adulte) trial compared CHVP (cyclophosphamide, adriamycin, etoposide, and prednisolone) regimen plus interferon with the same chemotherapy regimen combined with six infusions of rituximab and interferon for the same time period (R-CHVP-I arm), leading to a significant improvement in event-free survival in the R-CHVP-I arm ( *P* = 0.001). The estimates for 5-year OS were not statistically different; however, in those patients with the highest FLIPI score (n = 162), outcome was shown to be significantly different for both 5-year event-free survival ( *P* = 0.001) and OS ( *P* = 0.025) ^25^. Since these four published randomized trials, other prospective trials compared different chemotherapy regimens associated with rituximab in first-line FL treatment. Rummel *et al*. reported a randomized trial comparing R-CHOP with a rituximab-bendamustine regimen ^26^. Median PFS rates were 69.5 and 31.2 months in the rituximab-bendamustine and the R-CHOP groups ( *P* <0.001), respectively, and the latter showed no improvement of OS ^26^. It is important to note that this trial population included not only FL but also other indolent lymphoma. Moreover, the response rate with R-CHOP was lower than in the other published prospective trials. A randomized trial (FOLL05) was conducted by the Italian lymphoma intergroup (ILI) to compare the efficacy of eight doses of rituximab accompanied by six cycles of CHOP, eight cycles of CVP, or six cycles of fludarabine and mitoxantrone (FM). Follow-up was undertaken after a median period of 34 months, and PFS rates at 3 years were significantly different (52%, 68%, and 63%, respectively), but there was no difference in terms of OS ^27^. In total, 23 second malignancies were observed during follow-up, mainly in the rituximab plus FM (R-FM) arm. In conclusion, R-CHOP and R-FM were superior to R-CVP in time to treatment failure and PFS, but R-FM treatment was more toxic.

With a median follow-up of 7 years and an 83% 8-year OS rate, long-term follow-up of the FOLL05 trial confirms the favorable outcome of patients with advanced-stage FL treated with immunochemotherapy. Compared with those receiving R-CHOP, patients who initially received R-CVP had higher risks of progressing to lymphoma and requiring additional therapy ^28^.

More recently, the GALLIUM trial assessed the role of GA101 versus rituximab combined with CHOP, bendamustine, or CVP. The 3-year PFS (the main objective of the study) and the time to next treatment (TTNT) were better with GA101 compared with the rituximab arms ^15^. This was observed with the three chemotherapy regimens, even if GA101 and bendamustine seem to be associated with higher toxicity ^29^, probably because of T-cell decrease. Finally, in the phase 3 RELEVANCE trial ^30^, rituximab plus chemotherapy was compared with rituximab plus lenalidomide followed by maintenance monotherapy with rituximab in those patients who had not received any previous treatment for FL. Patients were randomly assigned to one of the two treatment schedules, and maintenance monotherapy with rituximab followed. PFS and complete response (confirmed or unconfirmed) at 120 weeks were the primary end points. In total, 1030 patients were included; the rates of confirmed or unconfirmed complete response at 3 months were comparable: 48% (95% CI 44–53) in the group receiving rituximab-lenalidomide and 53% (95% CI 49–57) in the group receiving rituximab-chemotherapy ( *P* = 0.13). The interim rates of PFS at 3 years were 77% (95% CI 72–80) and 78% (95% CI 74–82), respectively. Toxicity was different; there were higher rates of grade 3 to 4 neutropenia (32% versus 50%) and febrile neutropenia of any grade (2% versus 7%) in the R-CHOP group; conversely, patients in the rituximab-lenalidomide group had a higher percentage of grade 3 or 4 cutaneous reactions (7% versus 1%).

In conclusion, chemotherapy in FL is now systematically combined with anti-CD20 antibodies and has evolved to be the new standard of care for disseminated FL worldwide. CHOP and bendamustine were the most widely used chemotherapy regimens in 2018. Recent data have shown the possible advantage of GA101 combined with CHOP treatment as well as the possibility of obtaining similar results with chemo-free regimens. Longer follow-up is needed to obtain mature data regarding toxicity.

## Maintenance treatment

In treatment-naïve patients as well as in those with relapsed/refractory disease, rituximab has also been shown to extend the duration of response. The aim of the PRIMA trial was to assess the benefit of 2 years of MR following first-line treatment in FL patients receiving a rituximab-chemotherapy regimen; 1217 patients received one of three non-randomized, commonly used immunochemotherapy induction regimens; following this, 1019 patients attaining a complete or partial response were given MR therapy for 2 years or observation. PFS was the primary end point; after a median period of 36 months of follow-up, the substantial PFS benefit seen with MR (74.9% in the group receiving MR and 57.6% in the observation group) did not translate to a statistically significant benefit for OS. A longer period of follow-up may thus be needed. Toxicity was manageable. TTNT was also improved in the maintenance arm ^31^. These results were confirmed with a 10-year follow-up; despite the lack of OS benefit, more than half the patients in the rituximab arm remain free of disease progression and have not required new anti-lymphoma treatment beyond 10 years ^32^.

## Transformation

At the time of transformation, histology is most frequently diffuse large B-cell lymphoma (80%) and less common are composite lymphomas (14%) or lymphomas similar to morphologically high-grade B-cell lymphomas (6%). Since the majority of FLs have the t(13–17) and MYC translocation is a common event driving transformation to aggressive lymphoma, a majority of transformed FLs are double-hit lymphomas and are classified as high-grade B-cell lymphoma with MYC and BCL2 or BCL6 translocations or both. In the PRIMA trial, 37% of the patients who progressed in the 2 years had transformed non-Hodgkin lymphoma. Altered performance status, anemia, high lactate dehydrogenase level, “B” symptoms, histological grade 3a, and high FLIPI scores at diagnosis were identified as risk factors. Estimated median OS for the patients with histological transformation was low (3.8 years). When these patients received autologous stem cell transplantation, outcomes were improved ^33^.

## New drugs in follicular lymphoma

During the last decade, development of biological knowledge and use of targeted drugs have offered new perspectives in the treatment of FL. Most of the data come from phase 2 trials in a relapse setting and few of them have been introduced in first line. These new drugs include immunoconjugated antibodies, proteasome inhibitors, inhibitors of B-cell receptor pathway, and checkpoint inhibitors ^34^. Immunoconjugates are made of monoclonal antibodies associated with a cytotoxic drug entering the cell when the antibody binds its target. Inotuzumab ozogamicin (CMC-544) is a humanized anti-CD22 antibody conjugated with calicheamicin, a toxic agent that binds DNA leading to apoptosis. Polatuzumab vedotin is an anti-CD79b antibody conjugated with an anti-microtubule agent (monomethyl auristatin E). Both have shown promising results in association with rituximab in relapsed patients; the overall response rate was above 70% but there were hematological (inotuzumab ozogamicin) and neurological (polatuzumab vedotin) toxicities. In untreated patients, bortezomib, a proteasome inhibitor, has been used in association with rituximab and bendamustine with an estimated PFS at 3 years of 75% ^35^. Idelalisib, a phosphatidylinositol 3-kinase (PI3K) inhibitor, has been approved in FL in relapsed and refractory patients but its development in first line was stopped because of an increased risk of death, including *Pneumocystis carinii* and cytomegalovirus infections in patients who received idealisib combinations. Other PI3Ks such as copanlisib, duvelisib, or TGR-1202 are in development. Ibrutinib, a BTK inhibitor, has been evaluated in a front-line setting in association with rituximab with complete remission in 27% of the 60 patients ^36^. Venetoclax, a selective second-generation BCL-2 inhibitor, has been tested in a relapse setting with promising results. Front-line treatment is currently in evaluation. PD-1 expression is an escape mechanism for different hematologic malignancies. PD-1 inhibitors, such as pidilizumab and nivolumab, activity have recently shown activity in Hodgkin disease and more recently in other lymphomas, including FL, but are not use in front line.

## Conclusions

FL is an indolent lymphoma with different therapeutic approaches according to tumor burden. Better rates of remission and survival have been obtained since the introduction of rituximab in combination with chemotherapy and in maintenance therapy. More recently, the development of biological knowledge and the use of targeted drugs offer new therapeutic perspectives with chemo-free treatment strategies.
